# Dual‐Laser Optical Tweezers for Photothermal Analysis of Hybrid Microgels

**DOI:** 10.1002/advs.202511592

**Published:** 2025-11-25

**Authors:** Se‐Hyeong Jung, Chi Zhang, Nick Stauffer, Frank Scheffold, Lucio Isa

**Affiliations:** ^1^ Department of Materials ETH Zurich Vladimir‐Prelog‐Weg 1‐5/10 Zurich 8093 Switzerland; ^2^ Department of Physics Université de Fribourg Ch. Du. Musée 3 Fribourg 1700 Switzerland

**Keywords:** gold nanoparticles, hybrid microgels, optical tweezers, photo‐thermal actuation

## Abstract

Soft actuators that respond to external stimuli play a fundamental role in microscale robotics, active matter, and bio‐inspired systems. Among these actuators, photo‐thermal hybrid microgels (HMGs) containing plasmonic nanoparticles enable rapid, spatially controlled actuation via localized heating. Understanding their dynamic behavior at the single‐particle level is crucial for optimizing performance. However, traditional bulk characterization methods such as dynamic light scattering (DLS) provide only ensemble‐averaged data, thereby limiting analytical insights. Here, a dual‐laser optical tweezers approach is introduced for real‐time, single‐particle analysis of HMGs under controlled light exposure. Combining direct imaging and mean‐squared displacement (MSD) analysis, our method quantifies the precise laser power required for actuation and accurately tracks the particle size. The results are benchmarked against dual‐laser DLS, demonstrating comparable precision while offering the unique advantage of single‐actuator resolution. Thus, this method provides a robust platform for precise optimization of programmable actuators with applications in soft robotics, microswimmers, and biomedical devices.

## Graphical abstract

Dual‐laser optical tweezers unlock real‐time, single‐particle resolution of light‐driven actuation. Direct visualization and precise quantification of size changes under varying laser intensity provide new insights, overcoming limitations of conventional microscale actuator analysis.

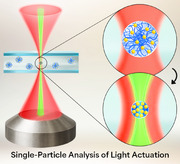

## 1 Introduction

Soft actuators enable programmable shape transformations and mechanical responses to external stimuli, making them fundamental components of adaptive materials.^[^ ^1^, ^2^, ^3^, ^4^ ^]^ Their high deformability and responsiveness provide a key advantage over rigid actuators, particularly at the microscale, where low energy consumption and precise adaptation to stimuli such as light, temperature, pH, and magnetic fields are crucial.^[^ ^5^, ^6^, ^7^ ^]^ These properties have been leveraged in diverse microscale applications, including thermo‐responsive microvalves for fluidic control, light‐driven microswimmers for targeted transport, and soft micromechanical oscillators for sensing.^[^ ^8^, ^9^, ^10^, ^11^, ^12^ ^]^

Among microscale soft actuators, microgels (MGs) stand out due to their reversible size change in response to a broad range of external stimuli. Microgels are defined as “particles of gel of any shape with an equivalent diameter of ≈0.1 to 100 µm” according to IUPAC.^[^ ^13^ ^]^ These cross‐linked polymer networks undergo swelling/deswelling as a function of environmental conditions, such as temperature, ionic strength, or pH. Adaptation to different stimuli therefore, allows the dynamic tuning of size, shape, porosity, mechanical properties, and chemical composition of individual particles,^[^ ^14^, ^15^, ^16^, ^17^, ^18^, ^19^, ^20^, ^21^ ^]^ leading to self‐regulated, programmable actuation without complex external control systems.^[^ ^22^, ^23^, ^24^, ^25^ ^]^ A prominent example is poly(*N*‐isopropylacrylamide) (PNIPAM)‐based microgels, which undergo a sharp, reversible phase transition from a swollen, hydrophilic state to a collapsed, hydrophobic state above PNIPAM's lower critical solution temperature (LCST, ≈32 °C).^[^ ^16^, ^20^, ^26^, ^27^ ^]^ This sharp transition is characterized by volumetric change of the microgel at the so‐called volume phase transition temperature, or VPTT, and makes PNIPAM microgels highly effective for precise shape transformations in microscale soft actuation.

Although PNIPAM‐based microgels exhibit efficient and reversible actuation, bulk thermal activation via global temperature modulations limits response speed, spatial precision, and independent control of individual actuators. To address this challenge, researchers have developed photothermal hybrid microgels (HMGs) by incorporating plasmonic nanoparticles capable of efficiently absorbing light and generating localized heat via surface plasmon resonance.^[^ ^28^, ^29^, ^30^, ^31^ ^]^ Among these, gold nanoparticles (AuNPs) are widely used due to their strong plasmonic response at 532 nm, enabling rapid heating and selective microgel collapse.^[^ ^32^ ^]^ This light‐driven actuation strategy has unlocked new possibilities in microscale systems, such as photo‐controlled Pickering emulsion,^[^ ^29^ ^]^ nanomechanical oscillators,^[^ ^33^ ^]^ and plasmonic self‐assembled materials.^[^ ^34^ ^]^

To fully exploit photothermal HMGs in soft actuation, precise characterization of their responses is essential. Previous studies have demonstrated the possibility of estimating the VPTT of thermo‐responsive microgels using differential scanning calorimetry (DSC)^[^ ^35^ ^]^ and UV–vis spectroscopy, both during irradiation^[^ ^36^ ^]^ and via temperature‐controlled measurements.^[^ ^29^ ^]^ While these approaches enable a quantification of the temperature response, they do not provide direct information on particle size as a function of temperature as modulated by a light source. To this end, an advanced dual‐laser dynamic light scattering (DLS) technique^[^ ^32^ ^]^ has been proposed to provide in situ size measurements under laser irradiation. Here, one laser is used to excite the metallic nanoparticles and cause local heating, while the other one probes the size changes. However, bulk DLS analysis is fundamentally limited by averaging effects, global heating artifacts dependent on particle concentration, and the assumption of a uniform light absorption and heat conversion for all HMGs. Thus, heterogeneous particle behavior and localized responses remain unresolved, particularly in mixed or polydisperse actuator systems.

To overcome these limitations, we introduce a dual‐laser optical tweezers method that enables real‐time analysis of single‐particle HMG response under controlled light exposure. By directly visualizing HMG size changes through image tracking and extracting dynamic properties through mean‐squared displacement (MSD) analysis, our approach precisely quantifies the laser power required for particle collapse, resolving variations that ensemble techniques cannot detect. To benchmark the consistency of our dual‐laser optical tweezers approach, we compare it with dual‐laser DLS. For this comparison, we extend the DLS measurements from previous studies to a larger scattering angle of 90°^[^ ^32^ ^]^ and, by accounting for the drift induced by laser radiation pressure, we achieve improved accuracy. Direct comparisons confirmed that both methods yield similar results, validating our optical tweezers data. However, optical tweezers provide additional crucial advantages, enabling real‐time visualization and precise characterization under conditions closely resembling practical applications. Beyond HMGs, this powerful single‐particle analytical method can be broadly applied to other microscale systems such as colloidal actuators, light‐driven microswimmers, and reconfigurable soft materials, opening new possibilities in responsive material design.

## 2 Results and Discussion

**Figure 1:**
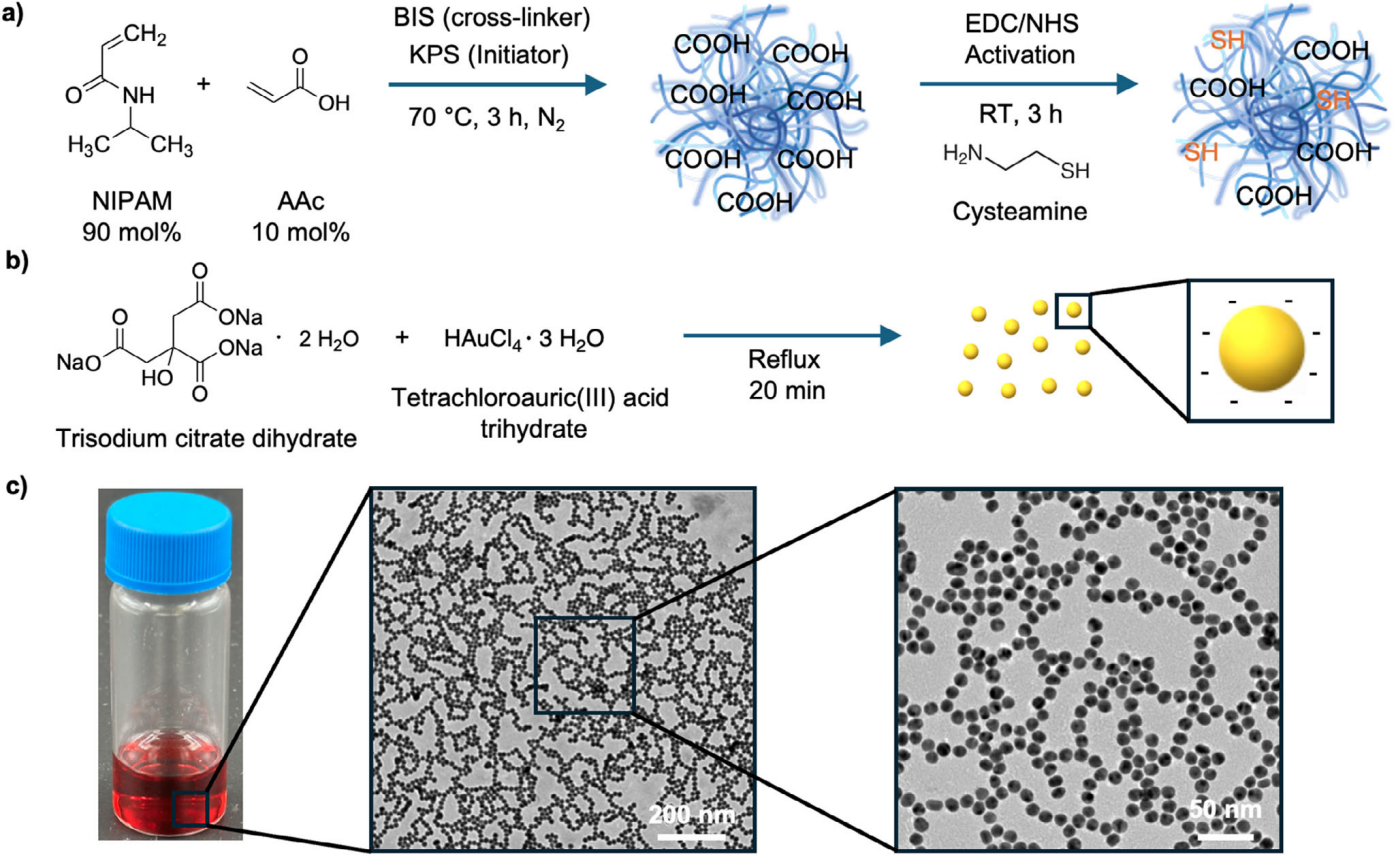
Schematic illustration of the synthesis and modification steps for microgels a) and AuNPs b). c) shows an image of AuNPs dispersion in water, along with TEM images of the synthesized AuNPs in the dry state.

A narrow plasmonic absorption band and a large volumetric response are central to the precise actuation of HMGs. We therefore first systematically optimized our particle systems. Initially, we synthesized PNIPAM‐co‐poly(acrylic acid) (PAAc) MGs via precipitation polymerization (**Figure** **1**a). The COOH groups (of PAAc) in the MGs, which confer negative charges at pH ≈5.5–6 (Milli‐Q water),^[^ ^37^ ^]^ ensure colloidal stability. The characterization of these MGs showed a hydrodynamic radius (R_h_) of 756 ± 15 nm and a zeta potential of −14.2 ± 0.1 mV, confirming the incorporation of COOH groups.^[^ ^38^, ^39^ ^]^ The COOH groups were subsequently substituted with cysteamine through EDC/NHS coupling for activation.^[^ ^40^, ^41^ ^]^ The MG's zeta potential slightly increases and the size decreases with higher thiol (SH) content (from 12.5 mol% to 50 mol%), corresponding to a decrease in the number of COOH groups (Table S1, Supporting Information). This reduction in surface charge leads to lower size due to weaker electrostatic interactions and osmotic pressure changes.^[^ ^38^, ^39^ ^]^ Additionally, we quantified the COOH groups before and after modification with cysteamine via FT‐IR spectroscopy (Figure S1, Supporting Information).^[^ ^21^ ^]^ The results show that the incorporated amount of COOH is close to the theoretical values (e.g., ≈10.7 mol% before modification, and 5.9, 7.7, and 8.5 mol% after modification with cysteamine, respectively). Next, we synthesized AuNPs by reducing Au^3+^ salts with citrate according to the Turkevich method,^[^ ^42^, ^43^, ^44^ ^]^ as shown in Figure 1b. The resulting monodisperse AuNPs exhibited the characteristic red color (Figure 1c), indicating a stable dispersion, with a zeta potential of −33.0 ± 2.4 mV. TEM images revealed a uniform particle size of 12 ± 0.2 nm (Figure 1c; Figure S2, Supporting Information), compatible with DLS measurements indicating a R_h_ of 18 nm, accounting for the core and surrounding solvation layer, adsorbed ions, or stabilizing agents.^[^ ^45^ ^]^ (The DLS and zeta potential measurements of the MGs and AuNPs are summarized in Table S1, Supporting Information).

**Figure 2:**
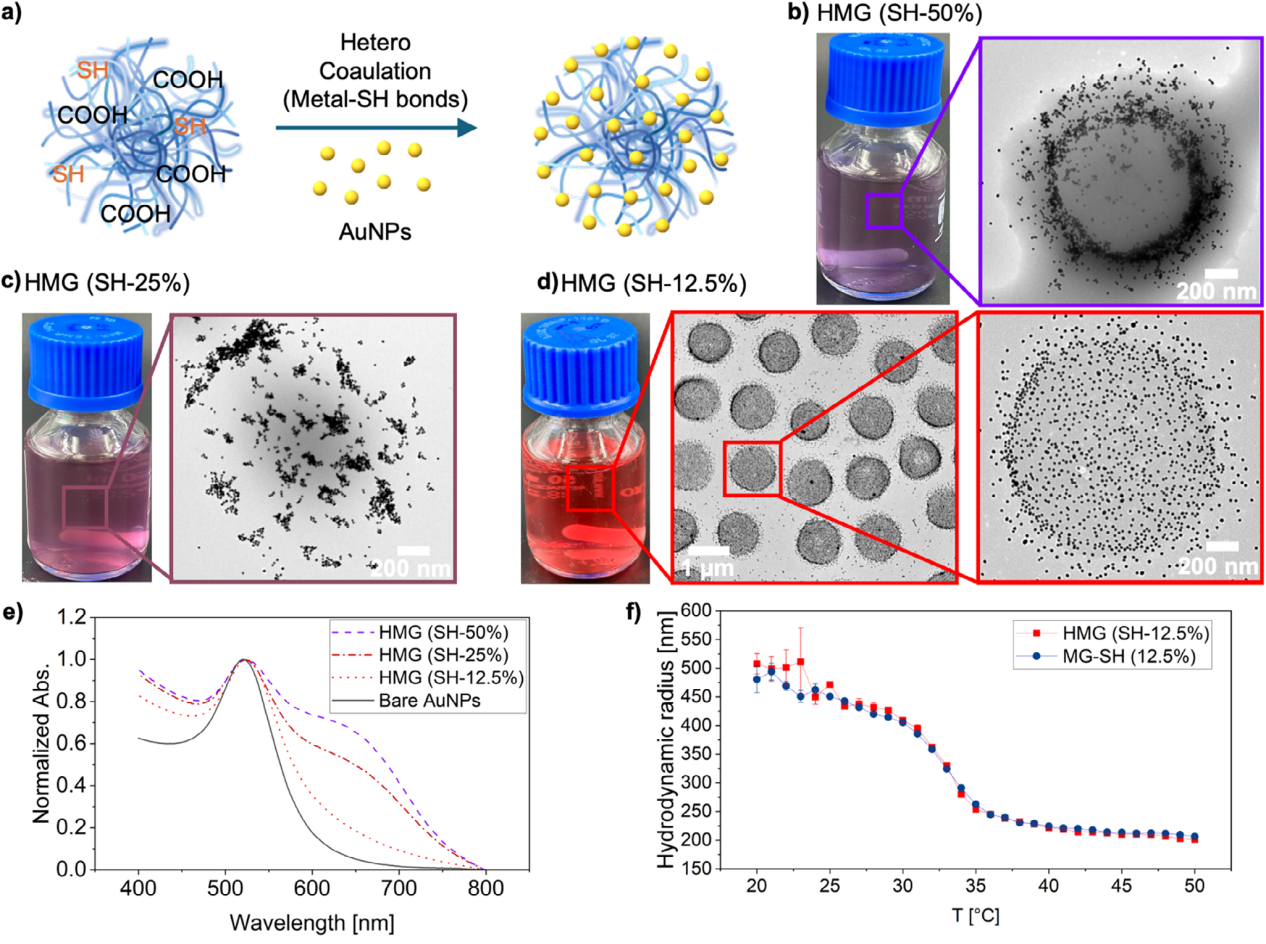
Schematic illustration of the fabrication process for HMGs a). Photographs of HMG dispersions along with their corresponding TEM images in the dry state are shown in b–d). e) UV–vis spectra of HMGs compared with bare AuNPs. f) T‐dependent hydrodynamic radius (R_h_) of HMGs and MGs with 12.5 mol% SH measured by DLS. All percentages are given in mol%.

Next, we prepared HMGs via hetero‐coagulation by mixing microgels and AuNPs, taking advantage of the strong binding affinity of SH groups toward metallic nanoparticles (**Figure** **2**a).^[^ ^46^, ^47^, ^48^ ^]^ The ratio between COOH and SH groups plays a crucial role in controlling aggregation behavior. Although elemental analysis showed slight deviations from the theoretical sulfur (S) content, the overall trend confirmed an increase with higher cysteamine concentrations (Table S2, Supporting Information). Together with FT‐IR analysis (Figure S1, Supporting Information), these results provide further evidence that the desired modification was successfully achieved. TEM images (Figure 2b–d) reveal that increasing SH content from 12.5 mol% to 25 mol% and 50 mol% leads to AuNP clustering within the microgels. The 12.5 mol% modification enables high loading of the AuNPs (> 1000 AuNPs per microgel, Figure S3, Supporting Information) while preventing aggregation (AFM images, Figure S4, Supporting Information) and is thus chosen for the subsequent analytical experiments. This trend is further reflected in bulk solution color changes, where higher SH concentrations lead to a visible darkening indicating particle clustering. We quantify these changes by means of UV‐Vis spectroscopy. The bare AuNPs show a sharp absorption peak at 521 nm, characteristic of a stable dispersion of highly monodisperse particles of this size (black curve in Figure 2e).^[^ ^44^ ^]^ HMGs with 12.5 mol% SH also exhibit a single peak at 521 nm with no shift, confirming the absence of aggregation. Increasing SH content instead leads to a redshift in the absorption spectra^[^ ^49^, ^50^ ^]^ and the emergence of a shoulder at higher wavelength with increase associated to NP clustering (Figure 2e). These results highlight the importance of balancing SH‐mediated AuNP binding with COOH‐driven electrostatic stabilization to maintain stable HMGs. To verify the importance of SH groups for efficient AuNP incorporation, we synthesized HMGs without SH modification as a negative control. TEM images revealed that in the absence of cysteamine modification, only small amounts of AuNPs are co‐localized with the MGs, with most particles freely dispersed in the medium (Figure S5, Supporting Information). This data confirms that SH modification is essential for high loading of AuNPs. The negative measured zeta potential of the HMGs also confirms that the HMGs possess sufficient charge to remain stable in aqueous media (Table S3, Supporting Information). Finally, DLS measurements with a temperature bath showed that MG‐SH 12.5 mol% and HMGs have an identical temperature response and collapse around the same volume phase transition temperature (VPTT, 30–32 °C, Figure 2f), indicating that the AuNPs do not strongly affect the polymer network response and architecture.

**Figure 3:**
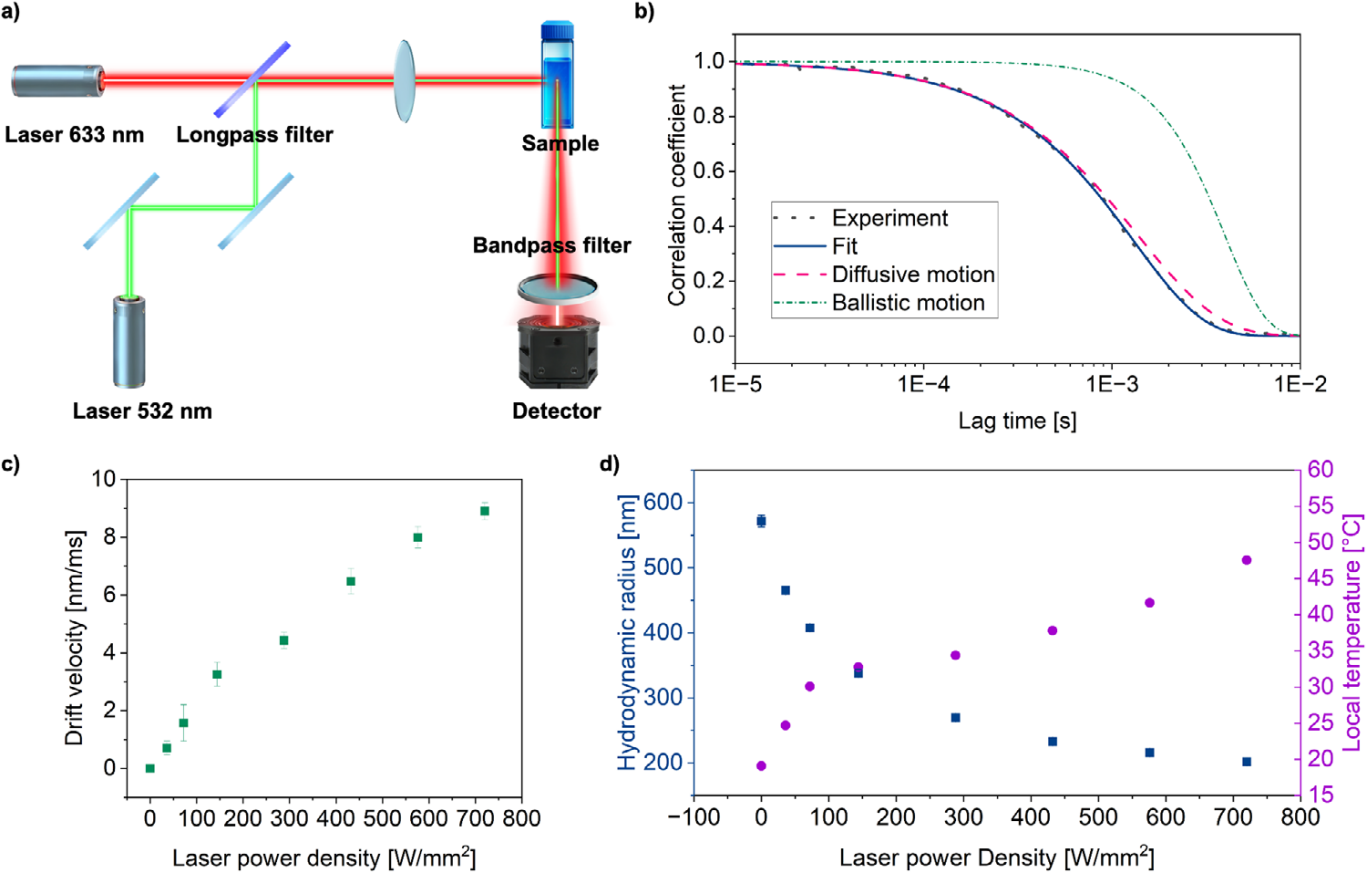
a) Experimental setup used for photothermal responsiveness analysis using dual‐laser DLS. b) Example of scattering time autocorrelation function, displaying experimental data, a purely diffusive fit, and a fit with an additional ballistic motion component. Inclusion of ballistic motion is essential for accurately fitting the data. c) Drift velocity extracted from the fits, showing the increase with laser power. d) R_h_ obtained from the fitted data as well as estimated local temperature versus laser power density. The estimated T is derived by comparing the measured R_h_ values from T‐batch DLS (Figure 2f).

Next, we adopted the recently introduced dual‐laser DLS approach developed by Lehmann et al.^[^ ^32^ ^]^ (**Figure** **3**a). In their method, a red laser (633 nm) was employed for scattering, while a green laser (532 nm) was used to induce photothermal shrinkage. Although they observed a clear size reduction of HMGs with increased green laser power, an anomalous rise in diffusivity was noted at higher scattering angles (q > 0.013 nm^−1^, ≈60°). They attributed this anomaly to inhomogeneous scattering and recommended limiting measurements to lower angles. Our investigation indicated that the anomalous diffusivity arises from an additional ballistic contribution induced by laser radiation pressure, i.e., the direct momentum transfer of photons driving a weak directional drift of the particles. A rough estimate supports this interpretation: for a hybrid microgel of radius ≈300 nm containing ≈1000 AuNPs (≈12 nm diameter, absorption cross section ≈40–60 nm^2^ at 532 nm),^[^ ^51^, ^52^ ^]^ illumination at 300 W mm^−2^ yields a radiation force of ≈50 fN, corresponding to a drift velocity of ≈9 nm ms^−1^. To account for this effect, we extended the standard DLS analysis by including a ballistic term in the correlation function, allowing both diffusive and drift contributions to be captured simultaneously. The theoretical basis of these expressions and the corresponding fitting procedure have been derived in detail in references.^[^ ^53^, ^54^ ^]^ Using this approach, we could reliably fit the measured correlation functions (Figure 3b) and quantify the drift velocity, which increased proportionally with the green laser power (Figure 3c), thereby confirming radiation pressure as the origin of the anomaly. Importantly, once this ballistic artifact was corrected for, accurate hydrodynamic radius (R_h_) measurements were obtained even at a 90° scattering angle. This improved analysis clearly confirmed that the HMGs exhibit a steady decrease in size with increasing laser intensity (Figure 3d), closely matching the temperature‐controlled DLS measurements (Figure 2f). From these corrected data, we were able to reliably extract the local temperature changes within the HMGs as a function of green laser power density (Figure 3d). Importantly, this analysis assumed that the viscosity of the surrounding medium remained constant and was unaffected by local heating, closely aligning with traditional temperature‐controlled batch DLS measurement (Figure 2f). For comparison, we also performed calculations assuming that the local temperature around the HMGs changes, thus affecting the medium's viscosity. These results are presented separately in Figure S6 (Supporting Information).

**Figure 4:**
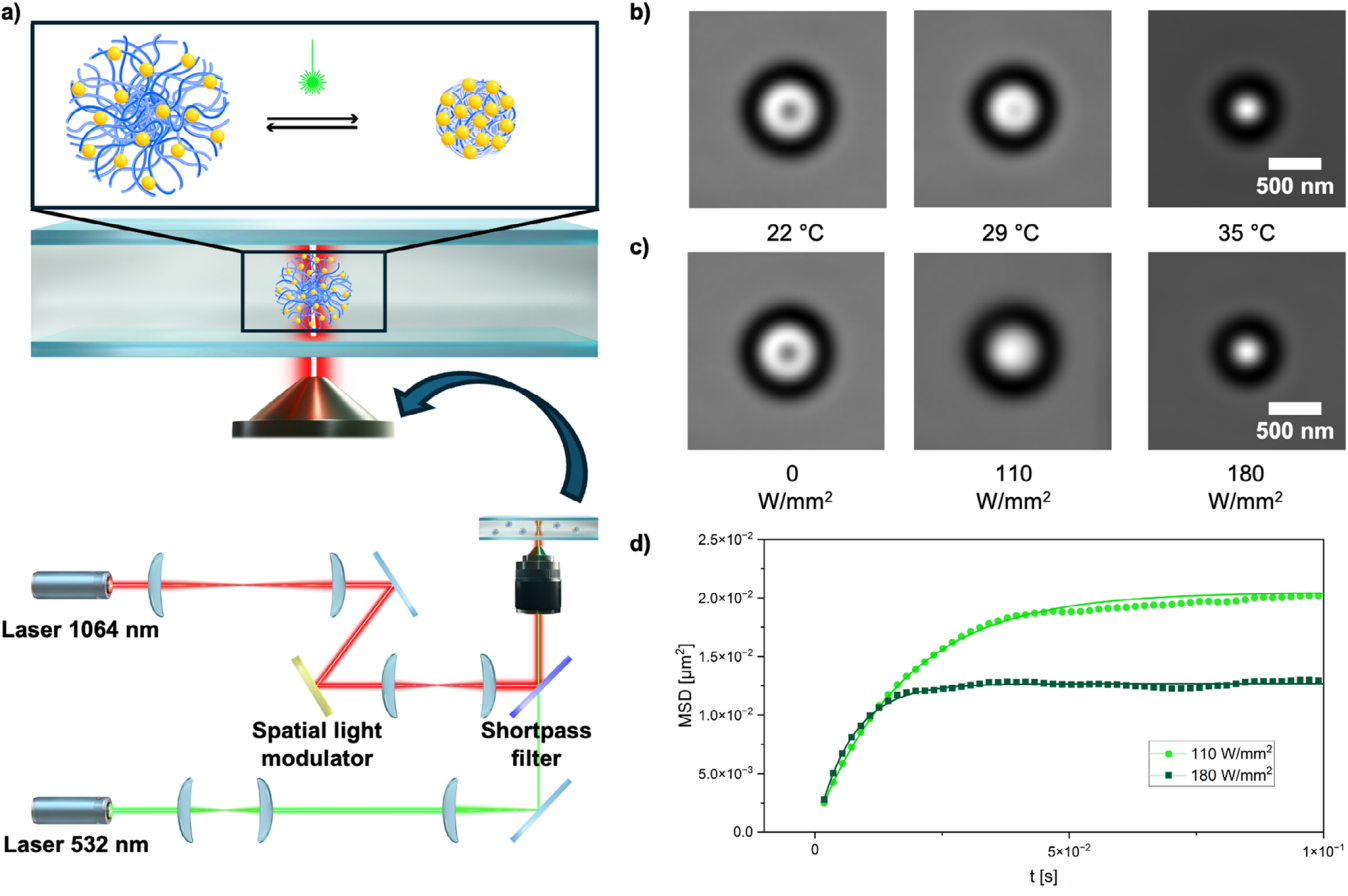
Illustration of the experimental setup for the two‐laser optical microscopy a). b) Optical micrograph at three different temperatures as examples, and c) optical micrographs at three different green laser powers measured at 22 °C. d) Mean square displacement (MSD) data (symbols) and corresponding fits (solid lines) at two laser powers (110 and 180 W mm^−2^).

To investigate the photothermal responsiveness of HMGs at the single‐particle level, we developed a dual‐laser optical tweezers method, where individual HMGs were trapped by an NIR laser (1064 nm) and simultaneously irradiated with a green laser (532 nm) (**Figure** **4**a). The HMGs are highly homogeneous, as confirmed TEM (Figure 2d), and the particles were selected randomly within the optical trap's field of view without pre‐screening to avoid selection bias. Initially, we trapped a single HMG and then gradually increased the green laser intensity until a clear collapse of the particle was visually confirmed (Video S1, Supporting Information). To accurately quantify these size changes, we first established a temperature calibration by imaging the same trapped HMG at controlled temperature (without green laser irradiation). Specifically, we recorded and averaged 1000 frames per particle at each known temperature within the optical trap; representative particle images at three different temperatures are shown in Figure 4b. From these images, we extracted the radial pixel intensity profiles from the particle center outward, creating a characteristic calibration profile at each temperature (representative examples in Figure S7, Supporting Information). Subsequently, we repeated the imaging under green laser irradiation (plasmonic heating) using an identical imaging protocol (Figure 4c). These images were then systematically compared against the full set of calibration profiles, and the temperature yielding the smallest mean residual error was selected as the estimated local temperature (representative examples in Figure S8, Supporting Information).^[^ ^55^ ^]^ The local temperatures and corresponding radius at various initial temperatures (22, 25, and 28 °C) are summarized in Table S4 (Supporting Information).

**Figure 5:**
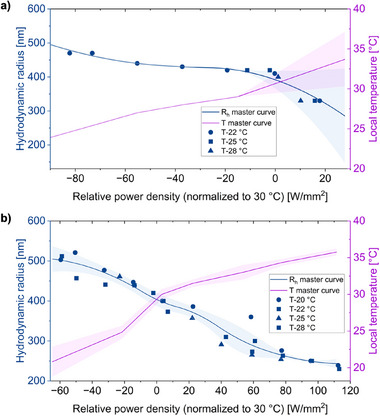
Estimation of R_h_ and local temperature of HMGs using a) image analysis and b) MSD‐based calculations. R_h_ (left y‐axis) and local temperature (right y‐axis) are plotted as a function of relative power density. Data obtained at different initial (ambient) temperatures were normalized to a common reference particle size of 409 nm at 30 °C (near the VPTT), corresponding to zero relative power density. Solid lines represent the master curves (quadratic‐spline interpolation for R_h_ and temperature), and shaded regions indicate the error range. A detailed description of the calculation and data‐processing procedure is provided in the Supporting Information.

The measured particle sizes obtained at different ambient temperatures were first aligned along the laser power‐density axis by converting them to relative power density, defined as the difference between the applied power and that required to reach the reference particle size of 409 nm at 30 °C (determined from temperature‐dependent DLS measurements, Figure 2f). This normalization, corresponding to the onset of the VPTT of the hybrid microgels (30–32 °C), enables direct comparison across different temperature conditions. The data lead to master curves for both R_h_ and the corresponding local temperature (**Figure** **5**a). These master curves clearly show a reproducible decrease in particle size and an increase in local temperature with rising relative power density, reflecting the progressive deswelling of the microgels under enhanced optical heating. To further validate the single‐particle analysis, we synthesized additional hybrid microgels composed of 50 mol% poly(*N*‐n‐propylacrylamide) (PNnPAM) and 50 mol% PNIPAM (loaded with the same amount of AuNPs without aggregation, Figure S9, Supporting Information), which exhibit a lower VPTT compared to the HMGs composed of 100 mol% PNIPAM (≈23–25 °C, Figure S9b, Supporting Information). We then compared the collapse behavior of both hybrid microgels using optical tweezers (Figure S9c, Supporting Information) in a heterogeneous environment containing a 1:1 mixture of the two HMG types. In these experiments, we determined the minimum laser power density required to trigger collapse at the single‐particle level. The PNnPAM 50 mol% HMG collapsed at ≈50 W mm^−^^2^, whereas the PNIPAM 100 mol% HMG required ≈160 W mm^−^^2^. These results were consistent across multiple cycles, demonstrating both reproducibility at individual particles and robustness of our single‐particle characterization approach. Together, these findings provide strong evidence that the methodology reliably captures heterogeneous particle behavior and actuator resolution at the single‐particle level.

While image analysis can be used to check quickly VPTT, it has practical limitations at temperatures (and correspondingly sizes) near and above the VPTT ≈30–32 °C due to limited variations in particle contrast as a function of temperature after deswelling (an example in Figure S8, Supporting Information with 180 W mm^−2^).

To extend reliable size estimation beyond the VPTT, we extracted the particle size from MSD‐based tracking analysis using particle trajectories recorded at high frame rates (560 fps, 10 000 frames per measurement). The MSD curves were fitted using Equation (1):^[^ ^56^ ^]^

(1) $$\begin{array}{c} <x^{2}\left(t\right)>=\frac{k_{B}T}{k}\left(1-e^{-2\mathrm{kt}/\gamma }\right) \end{array}$$

where k_B_ is the Boltzmann constant, T is absolute temperature, k is the trap stiffness, and γ is the drag coefficient. From these MSD fits (example shown in Figure 4d),^[^ ^57^ ^]^ we extracted the drag coefficient γ, subsequently determining the hydrodynamic radius R_h_ via Stokes–Einstein relation (Equation (2)) with η the medium viscosity:

(2) $$\begin{array}{c} \gamma =6\pi \eta R \end{array}$$

We chose a constant viscosity at ambient temperature for calculations, as this assumption yielded particle size data closely matching the temperature‐controlled DLS results (Figure 2f), suggesting minimal ambient viscosity changes around heated HMGs (raw data in Table S5, Supporting Information). This assumption is also consistant with the blinking behavior observed around the VPTT (30–32 °C, Video S2, Supporting Information), likely reflecting dynamic equilibrium between plasmonic heating and environmental cooling.^[^ ^58^, ^59^, ^60^ ^]^ Additional MSD analysis accounting for local viscosity changes due to localized heating are presented in Table S6 (Supporting Information). Following the same normalization procedure as in the image analysis, MSD datasets collected at initial temperatures of 20, 23, 25, and 29 °C were aligned to the common reference size of 409 nm (T = 30 °C). The resulting master curves (Figure 5b) clearly give a relation with R_h_ and local temperature as function of relative power density. Importantly, the MSD‐tracking analysis precisely captures trends even at temperatures above the VPTT, where the direct image‐based size analysis becomes unreliable due to limited changes in visual contrast.

## 3 Conclusion

In summary, our dual‐laser optical tweezers method enables direct visualization and quantitative assessment of single‐particle photothermal responses in hybrid microgels. By combining image analysis and MSD‐based tracking, we precisely determine particle size as a function of local temperature changes induced by defined laser irradiation. These measurements provide fundamental input parameters for future engineered actuation of HMG systems, and help identify reliable, reproducible building blocks for functional assemblies. For example, they could quantify temperature response for microswimmers whose propulsion depends on reversible volume changes, for soft robotic elements that require predictable, reversible actuation thresholds, and for biomedical carriers, which would benefit from sharp, reproducible transitions to minimize dosing variability.^[^ ^22^, ^23^, ^61^ ^]^ By resolving these properties at the single‐particle level, our approach complements bulk techniques and establishes a methodological platform for rational material selection in future microscale actuator systems. Looking forward, integrating this framework with mechanical property measurements (e.g., stiffness determination via optical tweezers analysis) could substantially extend its analytical reach toward adaptive and mechanically responsive micro‐materials.^[^ ^62^ ^]^

## Conflict of Interest

The authors declare no conflict of interest.

## Supporting information



Supporting Information

Supplemental Video 1

Supplemental Video 2

## Data Availability

The data that support the findings of this study are available from the corresponding author upon reasonable request.
