# Supplementary material for: Dual‐Laser Optical Tweezers for Photothermal Analysis of Hybrid Microgels
Source: Adv Sci (Weinh). 2025 Nov 25;13(7):e11592. doi: 10.1002/advs.202511592 (PMC12866740; doi:10.1002/advs.202511592)
Supplement: Supplementary file 1 — Supporting Information [file ADVS-13-e11592-s002.docx]

Dual-Laser Optical Tweezers for Photothermal Analysis of Hybrid Microgels

*Se-Hyeong Jung^†^*, Chi Zhang^†^, Nick Stauffer, Frank Scheffold, Lucio Isa**

**Materials**

# *N*-isopropylacrylamide (NIPAM) and *N*-n-propylarylamide (NnPAM) were purified by recrystallization from hexane prior to use. Inhibitor in acrylic acid is filtered using basic aluminum oxide in column before use. *N*,*N*’-methylenebisacrylamide (BIS, ≥99%), acylic acid (AAc, 99%), potassium persulfate (KPS, ≥99%), 1-Ethyl-3-(3-dimethylaminopropyl)carbodiimide hydrochloride (EDC, ≥99%), *N*-Hydroxysuccinimide (NHS, 98%), Sodium citrate tribasic dihydrate (≥99%), Tetrachloroauric(III) acid trihydrate (HAuCl₄·3H₂O, ≥99%) Hexane (≥99%), Hydrochloric acid (HCl, 37%), nitric acid (70%), basic aluminium oxide (99.99%), NIPAM (97%, stabilized with MEHQ) were purchased from Sigma-Aldrich. Dialysis tubing (Standard xRC, 12-14 and 3.5 kDa MWCO) was received from Spectra/Por^®^. Milli-Q water was prepared using a Milli-Q ultrapure water purification system (Merck Millipore, 18.2 MΩ·cm). Cysteamine (99%) was obtained from Fluorochem Ltd. 4-(2-sulfonatoethyl)morpholin-4-ium buffer (MES buffer, 1.0 M) was purchased from Thermo Fisher scientific. Carbon-coated copper grids (Cu 400 mesh) were obtained from Quantifoil. NnPAM (≥98%, stabilized with MEHQ) was purchased from TCI.

**Experiments and Synthesis**

**Microgel synthesis**

Microgel synthesis was performed in a 250 mL round-bottom flask. The standard microgel formulation contained NIPAM (0.764 g, 6.75 mmol, 90 mol%) and AAc (51.4 µL, 0.75 mmol, 10 mol%), together with BIS (17.3 mg, 0.113 mmol, 1.5 mol% based on total monomer), dissolved in 73 mL of Milli-Q water. For the plain PNIPAM microgels, NIPAM alone was used (0.849 g, 7.50 mmol, 100 mol%) without AAc. For the poly(*N*-n-propylacrylamide) (PNnPAM)-containing microgels, NnPAM (0.382 g, 3.375 mmol, 45 mol%) and an equal molar amount of NIPAM (45 mol%) were used together with AAc (10 mol%). All monomer solutions were degassed with nitrogen (N₂) for 30 min. Separately, KPS (0.035 g, 0.129 mmol) was dissolved in 2 mL Milli-Q water and degassed with N₂ for 45 min before initiation. The monomer + cross-linker mixture was gradually heated, reaching 70 °C for additional 15 min. The initiator solution was subsequently added to the monomer mixture, and the polymerization was started at 70°C and kept for 3 h. Afterward, the reaction mixture was filtered through filter paper (MN 615 1/4, Macherey-Nagel GmbH) to remove aggregated particles, then left overnight at RT to reduce further aggregation. The purified microgels underwent three cycles of centrifugation (14 000 rcf, 20 min, 20 °C), with resuspension in Milli-Q water between each centrifugation step. Finally, the microgels were dialyzed (12-14 kDa MWCO) against Milli-Q water for 7 days using dialysis tubing, with two changes a day of 5L Milli-Q water each time.

**Homopolymer PAAc**

AAc (205.6 µL, 3 mmol) was added to 29 mL of Milli-Q water. KPS (12.2 mg, 0.045 mmol) was dissolved in 1 mL of Milli-Q water. Both solutions were degassed with N_2_ for 45 min under stirring, and the monomer mixture was then heated to 70 °C. The initiator solution was added to the monomer solution, and the polymerization was carried out at 70 °C for 3 h. The resulting polymer mixture was purified by dialysis (3.5 kDa MWCO) against Milli-Q water for three days.

**Microgel modification with thiol**

10 mL of each microgel solution was transferred into separated reaction vials, and 1 mL of 1 M MES buffer was added to each vial (resulting in approximately pH 6.85 after mixing). To activate the carboxyl groups, 38.7 mg (0.2 mmol, 2 equivalents relative to AAc) of EDC and 23.5 mg (0.2 mmol, 2 equivalents relative to AAc) of NHS were sequentially added, and the mixtures were stirred at RT for 5 min. Subsequently, cysteamine was introduced into the solutions at varying amounts: 0.96 mg for 12.5 mol%, 1.93 mg for 25 mol%, and 3.86 mg for 50 mol% modifications. The reactions were allowed to proceed overnight continuous stirring at RT. Following the reaction, each modified microgel was purified by centrifugation (14 000 rcf, 20 °C, 20 min), repeated three times, with resuspension in Milli-Q water after each centrifugation step.

**Gold nanoparticle synthesis**

AuNPs were synthesized following the citrate reduction method, initially developed by Turkevich^1^ and later detailed by Kimling et al.^2^ Before synthesis, all glassware was thoroughly cleaned using aqua regia (a freshly prepared 3:1 mixture of concentrated hydrochloric acid and nitric acid), rinsed extensively with Milli-Q water and dried with N_2_. For synthesis, 98 mL of Milli-Q water was placed into a 250 mL two-neck flask equipped with a condenser and glass stoppers. Subsequently, 2 mL of a 50 mM (prepared by dissolving 33.9 mg HAuCl₄·3H₂O in Milli-Q water) was added, resulting in a final HAuCl_4_ concentration of 1 mM. The solution was heated until reflux (~120 °C). Then, 10 mL of a 38.8 mM sodium citrate solution (prepared by dissolving 114 mg trisodium citrate dihydrate in water) was rapidly added, and the stopper was replaced with a clean one. The reaction mixture changed color to dark red (wine-red) within 2-3 min. After 20 minutes, heating was stopped, and the mixture was allowed to cool to RT under continuous stirring. The AuNP suspension was stored at 4 °C for future use.

**Fabrication of hybrid microgels**

Hybrid microgels were fabricated using simple hetero coagulation. Specifically, 0.2 mL of each microgel solution was diluted to 5 mL by adding Milli-Q water. Subsequently, 5 mL of the previously prepared AuNP solution was added to each diluted microgel solution. The mixtures were stirred overnight at RT. The following day, the mixtures were purified by centrifugation twice (8 000 rcf, 20 °C, 5 min each), and resuspended in Milli-Q water after each centrifugation step.

**Dynamic light scattering (DLS) with T-batch and zeta potential measurements**

R_h_ at single temperature (20 °C) was measured using DLS (ALV GmbH) and zeta potential measurement were conducted by Zetasizer NanoZS (Malvern). All samples, including microgels and AuNPs, were diluted appropriately in Milli-Q water prior to analysis. DLS measurements were performed at a scattering angle of 90°. Each sample was measured three times to ensure reproducibility. Temperature-dependent DLS measurements (T-dependent curves) for hybrid microgels and microgels modified with 12.5 mol% SH groups were performed using a Nanolab 3D DLS instrument (LS instruments). Measurements were taken at a scattering angle of 90°, spanning a temperature range from 20 °C to 50 °C in increments of 1 °C. At each temperature point, measurements were repeated three times, ensuring accurate determination of size variations as a function of temperature. The standard error of the mean (SEM) was calculated from these triplicate measurements with the equation (1):

$$\begin{aligned} SEM= \frac{standard deviation \left( SD \right)}{\sqrt{n}}\#\left( 1 \right) \end{aligned}$$

**Transmission electron microscopy (TEM)**

TEM was conducted using a JEM-1400 (JEOL) operated at an acceleration voltage of 120 kV. Samples were prepared by placing a droplet of suspension (10 µL) onto carbon-coated copper grids (quantifoil). The samples were allowed to air dry at RT prior to imaging. Images were captured under standard conditions to analyze and size distribution of nanoparticles and hybrid microgels.

**Atomic Force Microscopy (AFM)**

Samples for AFM characterization were prepared by deposing a droplet of microgel suspension onto plasma-treated silicon wafers and allowing them to air-dry overnight in a fume hood. The measurements were conducted the following day using an Icon Dimension instrument (Bruker) operating in tapping mode under ambient conditions (air-surface). Imaging parameters included a proportional gain of 6.8, integral gain of 1.18, amplitude setpoint at 787 mV, and a scanning area of 3 µm x 3 µm. Both height and phase contrast data were recorded for detailed morphological analysis.

**UV-Vis spectroscopy**

UV-Vis absorption spectra were recorded using a Cary 60 UV-Vis spectrophotometer (Agilent Technologies). Measurements were performed in a wavelength range of 400-800 nm at medium scan speed at 20 °C. Milli-Q water was used as a reference for background correction prior to each measurement. Samples were analyzed in standard quartz cuvettes.

**FT-IR measurements and calibration**

Freeze-dried (two night by Free zone 4.5 L, Labconco) plain PNIPAM microgels and poly(acrylic acid) (PAAc) homopolymer were weighed and physically mixed. The exact amounts of both polymers were recorded and used to calculate the mol% of PAAc in the mixtures. Four mixtures were prepared, targeting 5, 10, 25, and 50 mol% PAAc; the precise mol% values were calculated from the weighed masses and are reported in Figure S1(c). All the mixtures were dissolved in 1 mL of Milli-Q water and freeze-dried overnight. FT-IR spectra (Lyza 7000, Anton Paar, 48 scans, background-corrected) were recorded for each mixture. To construct the calibration curve, the intensity ratio (Eq. 2) was determined by dividing the absorbance of the COOH stretching vibration (C=O of acrylic acid) at 1711 cm⁻¹ by the absorbance of the amide C=O band of PNIPAM at 1637 cm⁻¹:

$$\begin{aligned} Ratio=\frac{I_{1711}}{I_{1637}}\#\left( 2 \right) \end{aligned}$$

This ratio was plotted against the molar fraction of PAAc in the mixtures, yielding the calibration curve shown in Figure S1(c). The linear regression resulted in the following equation (3), which was used to calculate the COOH content of PNIPAM–PAAc microgels:

$$\begin{aligned} r_{PAAc}=\frac{intensity ratio-0.122}{0.019}\#\left( 3 \right) \end{aligned}$$

**Elemental analysis (CHNS)**

Elemental analysis was performed to determine the sulfur (S) content of the microgel samples. Prior to measurement, samples were dried overnight using a freeze dryer. Approximately 1 mg of each dried sample was weighed and analyzed using a HEKAtech EuroVector CHNS-O Elemental Analyzer, operated under standard combustion conditions to fully convert samples into gaseous combustion products for precise elemental quantification. The theoretical sulfur content was calculated based on the molar ratio of cysteamine incorporated during the modification step relative to the total monomer composition, assuming complete conversion and incorporation efficiency. Experimental values were compared against theoretical expectations to evaluate the efficiency of microgel functionalization.

**Dual-laser dynamic light scattering (DLS)**

A custom dual-laser DLS setup was constructed in-house (**Fig. 3a**). A green laser (532 nm, Verdi, Coherent) was employed for actuation purposes, while a red laser (633 nm, He-Ne, Newport) served as the detection source. Measurements were performed at a 90° scattering angle. To prevent detector saturation, the green laser was filtered using a bandpass filter before reaching the detector. Post-measurement data analysis, including fitting of autocorrelation functions (single exponential decay with drift) and extraction of decay times, was conducted using MATLAB-based routines developed in the laboratory.

**Dual-laser optical tweezers**

A custom-built dual-laser optical tweezers system was employed to trap and analyze particles while simultaneously tracking their position and shape. A loosely focused 532nm laser (Cobolt, HÜBNER Photonics) was used to induce plasmonic heating in gold-nanoparticle–loaded microgels, while bright-field illumination was provided by a white LED. Time-lapse images of the trapped particles were recorded using a high-resolution Prime 95B sCMOS camera (Teledyne Photometrics), mounted on a Nikon Ti2 microscope with an objective Apo TIRF 100X (numerical aperture of 1.49). The image sequences were analysed with custom MATLAB scripts based on the shape (Raidal intensity profile) of the particle. Mean-square displacement (MSD) analysis was performed to quantify mobility under different heating conditions, providing insight into particle dynamics and local interactions at the single-particle level.

**Master curve construction and data processing for dual-laser optical tweezers**

All datasets obtained at different initial (ambient) temperatures were normalized to a common reference point corresponding to 30 °C, determined from DLS measurement (**Fig. 2f**, R_h_ at 30 °C = 409 nm). This reference was chosen because it marks the onset of the volume phase transition temperature (VPTT) of the hybrid microgels (30-32 °C) and represents the last temperature range where both imaging and MSD analyses yield stable, reproducible particle sizes. For each dataset, the laser power density (PD), defined as the laser power (W) divided by the illuminated area (mm^2^), required to reach this reference particle size was determined by linear interpolation (Eq. 4) between two experiment data points:

$$\begin{aligned} {PD}_{30 ^{\circ}C} ={PD}_{1}+\left( 409-R_{h,1} \right) \frac{{PD}_{2}-{PD}_{1}}{R_{h,2}-R_{h,1}}\#\left( 4 \right) \end{aligned}$$

The power-density axis was then shifted such that this reference power (Eq. 5) became zero for each dataset:

$$\begin{aligned} {PD}_{relative}= {PD}_{measured}-{PD}_{30 ^{\circ}C}\#\left( 5 \right) \end{aligned}$$

Here, negative values of PD_relative_ correspond to laser powers below the threshold for 30 °C, while positive values indicate powers above this reference. All shifted datasets were combined and binned along the PD_relative_ axis. For each bin, the mean hydrodynamic radius (Eq. 6) and standard deviation (Eq. 7) were calculated as:

$$\begin{aligned} \overline{R_{h}}= \frac{1}{n}\sum_{i=1}^{n} R_{h,i}\#\left( 6 \right) \end{aligned}$$

$$\begin{aligned} \sigma_{R_{h}}= \sqrt{\frac{\sum_{i=1}^{n} \left( R_{h,i}-\overline{R_{h}} \right)^{2}}{n-1}}\#\left( 7 \right) \end{aligned}$$

The averaged values were interpolated using a quadratic spline to generate a smooth master curve, while the upper and lower error limits were obtained from $\overline{R_{h}}{\pm\sigma}_{R_{h}}$. The same normalization and binning procedure was applied the corresponding estimated temperature data, using linear interplation to construct the tempearature master curve. This normalization aligns all datasets on a single relative scale, enabling direct comparison of particle-size evolution and local heating behavior across different starting temperatures while accounting for experimental variability.

**Preparation of buffer solution**

The buffer solutions were adjusted to an ionic strength of I = 10 mM. Formic-acid-based buffers at pH 3.98 (≈4) were prepared according to the procedure described by D. D. Perrin.^3^

**Supporting Data**

| **Sample** | **DLS at 20 °C (mean R_h_)** | **Zetapotential at 20 °C** |
| --- | --- | --- |
| **Microgel-COOH** | 756 ± 15 nm | -14.2 ± 0.1 mV |
| **Microgel-SH-50 mol%** | 501 ± 8 nm | -11.8 ± 0.6 mV |
| **Microgel-SH-25 mol%** | 521 ± 15 nm | -12.3 ± 0.6 mV |
| **Microgel-SH-12.5 mol%** | 538 ± 16 nm | -13.5 ± 0.2 mV |
| **AuNPs** | 18 ± 0 nm | -33 ± 2.4 mV |

**Table S1**. Mean hydrodynamic radius (R_h_) and zeta potential values determined by DLS and electrophoretic mobility measurements, respectively. All the measurements were performed in Milli-Q water. “±” indicates the standard error of the mean calculated from independent measurements.

| **Sample** | **Experimental S% (1st)** | **Experimental S% (2nd)** | **Average Experimental S%** | **Theoretical S%** |
| --- | --- | --- | --- | --- |
| **Microgel-SH-50 mol%** | 1.12 | 1.22 | 1.17 | 1.45 |
| **Microgel-SH-25 mol%** | 0.86 | 0.85 | 0.86 | 0.73 |
| **Microgel-SH-12.5 mol%** | 0.42 | 0.54 | 0.48 | 0.36 |

**Table S2**. Sulfur content (%) determined by elemental analysis (CHNS) for microgel samples. Two experimental measurements are shown along with their average and the theoretically calculated sulfur content based on initial reagent ratios.

| **Sample** | **Zetapotential at 20 °C** |
| --- | --- |
| **HMG (SH-50 mol%)** | -10.7 ± 0.4 mV |
| **HMG (SH-25 mol%)** | -12.9 ± 1.2 mV |
| **HMG (SH-12.5 mol%)** | -13.7 ± 0.7 mV |

**Table S3**. Zeta potential values of the hybrid microgels determined by electrophoretic mobility measurements. All the measurements were performed in Milli-Q water. “±” indicates the standard error of the mean calculated from independent measurements.

| **Laser power density (W/mm²)** | **R_h_ (nm)**  **T_ini._-22 °C** | **Local T (°C)**  **T_ini._-22 °C** | **R_h_ (nm)**  **T_ini._-25 °C** | **Local T (°C)**  **T_ini._-25 °C** | **R_h_ (nm)**  **T_ini._-28 °C** | **Local T (°C)**  **T_ini._-28 °C** |
| --- | --- | --- | --- | --- | --- | --- |
| **28** | 470 | 25 | 420 | 25 | 400 | 31 |
| **37** | 470 | 25 | 420 | 29 | 330 | 33 |
| **55** | 440 | 27 | 330 | 29 | ≥ T_VPTT_ | ≥ T_VPTT_ |
| **73** | 430 | 28 | ≥ T_VPTT_ | ≥ T_VPTT_ | ≥ T_VPTT_ | ≥ T_VPTT_ |
| **91** | 420 | 29 | ≥ T_VPTT_ | ≥ T_VPTT_ | ≥ T_VPTT_ | ≥ T_VPTT_ |
| **110** | 410 | 30 | ≥ T_VPTT_ | ≥ T_VPTT_ | ≥ T_VPTT_ | ≥ T_VPTT_ |
| **128** | 330 | 33 | ≥ T_VPTT_ | ≥ T_VPTT_ | ≥ T_VPTT_ | ≥ T_VPTT_ |

**Table S4**. Hydrodynamic radius (R_h_) and local temperature estimates of hybrid microgels (HMGs), determined by dual-laser optical tweezers and image analysis. Measurements were performed at three different initial heating plate temperatures. Values above or around volume phase transition temperature (≥ T_VPTT_) could not be precisely differentiated due to comparable radial intensity profiles after deswelling. Uncertainties (±1 °C) arise from the 1 °C calibration step size.

| **Laser power density (W/mm²)** | **R_h_ (nm)**  **T_ini._-20 °C** | **Local T (°C)**  **T_ini._-20 °C** | **R_h_ (nm)**  **T_ini._-23 °C** | **Local T (°C)**  **T_ini._-23 °C** | **R_h_ (nm)**  **T_ini._-25 °C** | **Local T (°C)**  **T_ini._-25 °C** | **R_h_ (nm)**  **T_ini._-29 °C** | **Local T (°C)**  **T_ini._-29 °C** |
| --- | --- | --- | --- | --- | --- | --- | --- | --- |
| **28** | 503 ± 11 | 20 | 512 ± 31 | 23 | 461 ± 19 | 25 | 420 ± 20 | 29 |
| **37** | 521 ± 23 | 20 | 457 ± 21 | 24 | 442 ± 16 | 25 | 373 ± 17 | 31 |
| **55** | 477 ± 22 | 23 | 441 ± 18 | 25 | Blinking | Blinking | Blinking | Blinking |
| **73** | 447 ± 19 | 25 | 439 ± 18 | 26 | 357 ± 21 | 32 | 310 ± 18 | 33 |
| **91** | 400 ± 18 | 30 | 400 ± 17 | 30 | 291 ± 15 | 33 | 300 ± 18 | 33 |
| **110** | 386 ± 16 | 31 | Blinking | Blinking | 265 ± 15 | 35 |  |  |
| **128** | Blinking | Blinking | Blinking | Blinking | 254 ± 16 | 35 |  |  |
| **146** | 360 ± 17 | 32 | 273 ± 18 | 34 |  |  |  |  |
| **165** | 276 ± 16 | 34 | 263 ± 18 | 35 |  |  |  |  |
| **183** | 250 ± 14 | 35 | 250 ± 17 | 35 |  |  |  |  |
| **200** | 239 ± 6 | 36 | 230 ± 6 | 37 |  |  |  |  |

**Table S5**. Mean-square displacement (MSD) tracking based data for HMGs, obtained by dual-laser optical tweezers analysis at four different initial temperatures. The analysis assumed that the ambient temperature was unaffected; thus, viscosity values corresponded to the respective initial temperatures. Near the volume phase transition temperature (VPTT), accurate MSD calculations were challenging due to particle blinking (**Video S2**), indicating non-equilibrium conditions arising from competing heating by the green laser and cooling by the surrounding medium. Above VPTT, stable MSD measurements and calculations were achievable. Reported uncertainties (± x nm) correspond to 95% fit confidence boundary.

| **Laser power density (W/mm²)** | **R_h_ (nm)**  **T_ini._-20 °C** | **Local T (°C)**  **T_ini._-20 °C** | **R_h_ (nm)**  **T_ini._-23 °C** | **Local T (°C)**  **T_ini._-23 °C** | **R_h_ (nm)**  **T_ini._-25 °C** | **Local T (°C)**  **T_ini._-25 °C** | **R_h_ (nm)**  **T_ini._-29 °C** | **Local T (°C)**  **T_ini._-29 °C** |
| --- | --- | --- | --- | --- | --- | --- | --- | --- |
| **28** | 508 ± 11 | 20 | 501 ± 30 | 23 | 450 ± 19 | 25 | 427 ± 20 | 29 |
| **37** | 484 ± 21 | 20 | 462 ± 21 | 23 | 441 ± 16 | 25 | 395 ± 18 | 31 |
| **55** | 501 ± 23 | 22 | 451 ± 18 | 24 | Blinking | Blinking | Blinking | Blinking |
| **73** | 479 ± 23 | 23 | 450 ± 18 | 24 | 402 ± 24 | 30 | 341 ± 20 | 33 |
| **91** | 445 ± 24 | 24 | 433 ± 18 | 27 | 342 ± 18 | 33 | 332 ± 20 | 33 |
| **110** | 437 ± 18 | 25 | Blinking | Blinking | 316 ± 18 | 33 |  |  |
| **128** | Blinking | Blinking | Blinking | Blinking | 304 ± 18 | 33 |  |  |
| **146** | 432 ± 20 | 28 | 337 ± 22 | 33 |  |  |  |  |
| **165** | 362 ± 21 | 32 | 326 ± 22 | 33 |  |  |  |  |
| **183** | 333 ± 19 | 33 | 312 ± 21 | 33 |  |  |  |  |
| **200** | 320 ± 8 | 33 | 290 ± 8 | 34 |  |  |  |  |

**Table S6**. Mean-squared displacement (MSD) tracking based data for HMGs, obtained by dual-laser optical tweezers analysis at four different initial temperatures. The ambient temperature was considered equal to the total hybrid microgel temperature, and thus, viscosity values were adjusted accordingly. Near the volume phase transition temperature (VPTT), accurate MSD calculations were challenging due to particle blinking (**Video S2**), indicating non-equilibrium conditions arising from competing heating by the green laser and cooling by the surrounding medium. Above VPTT, stable MSD measurements and calculations were achievable. Reported uncertainties (± x nm) correspond to 95% fit confidence boundary.


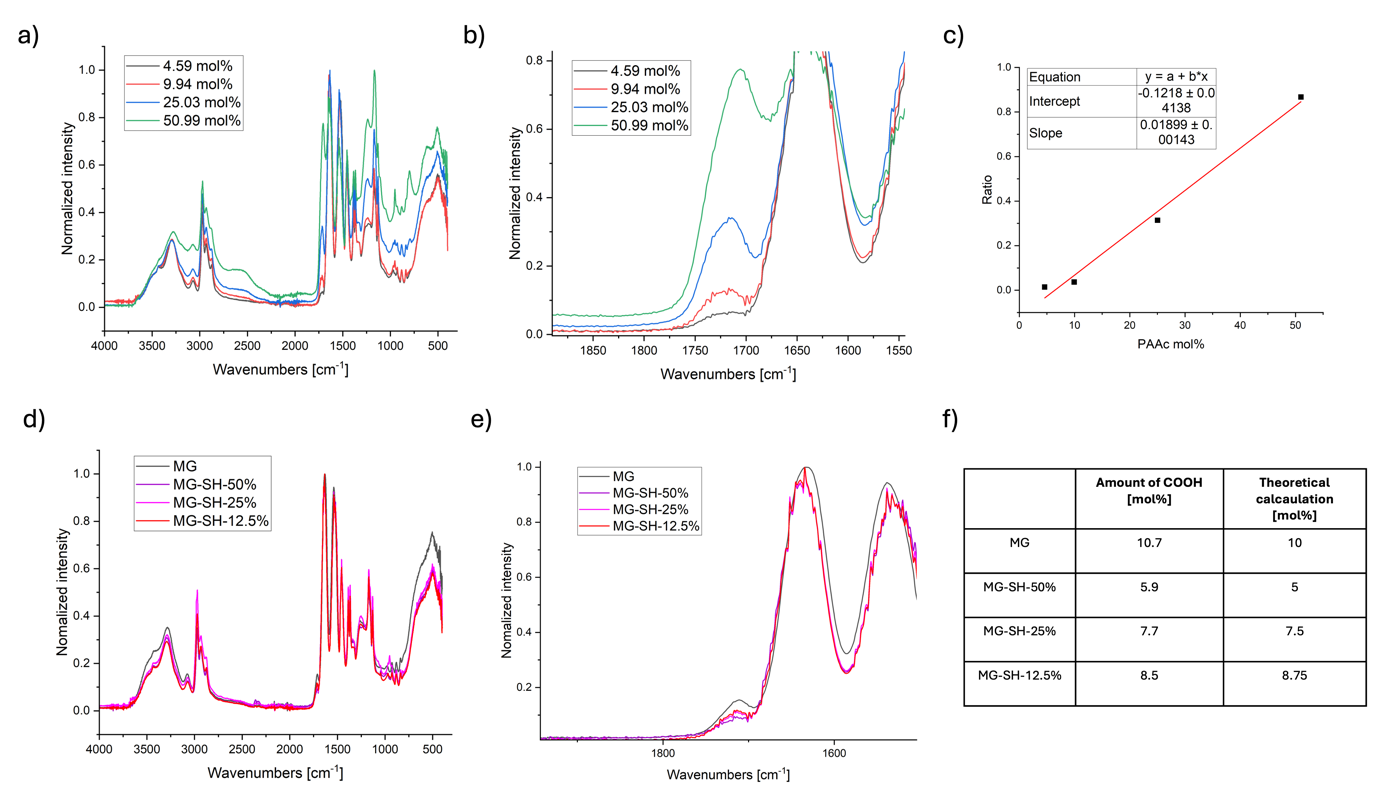


**Figure S1.** FT-IR spectra of PAAc homopolymer and PNIPAM microgels mixed at different molar ratios for calibration (a). Panel (b) shows the same spectra with a zoomed-in view for clarity. Panel (c) presents the calibration curve obtained by plotting the intensity ratio of the PAAc carbonyl peak (1711 cm⁻¹) to the PNIPAM amide I peak (1637 cm⁻¹) with the corresponding linear fit. FT-IR spectra of the microgels before and after modification with cysteamine are shown in (d), and panel (e) provides a zoomed-in view for improved visualization. Panel (f) summarizes the calculated COOH content of each microgel sample. All spectra were normalized to the PNIPAM amide I peak at 1637 cm⁻¹ to facilitate comparison.


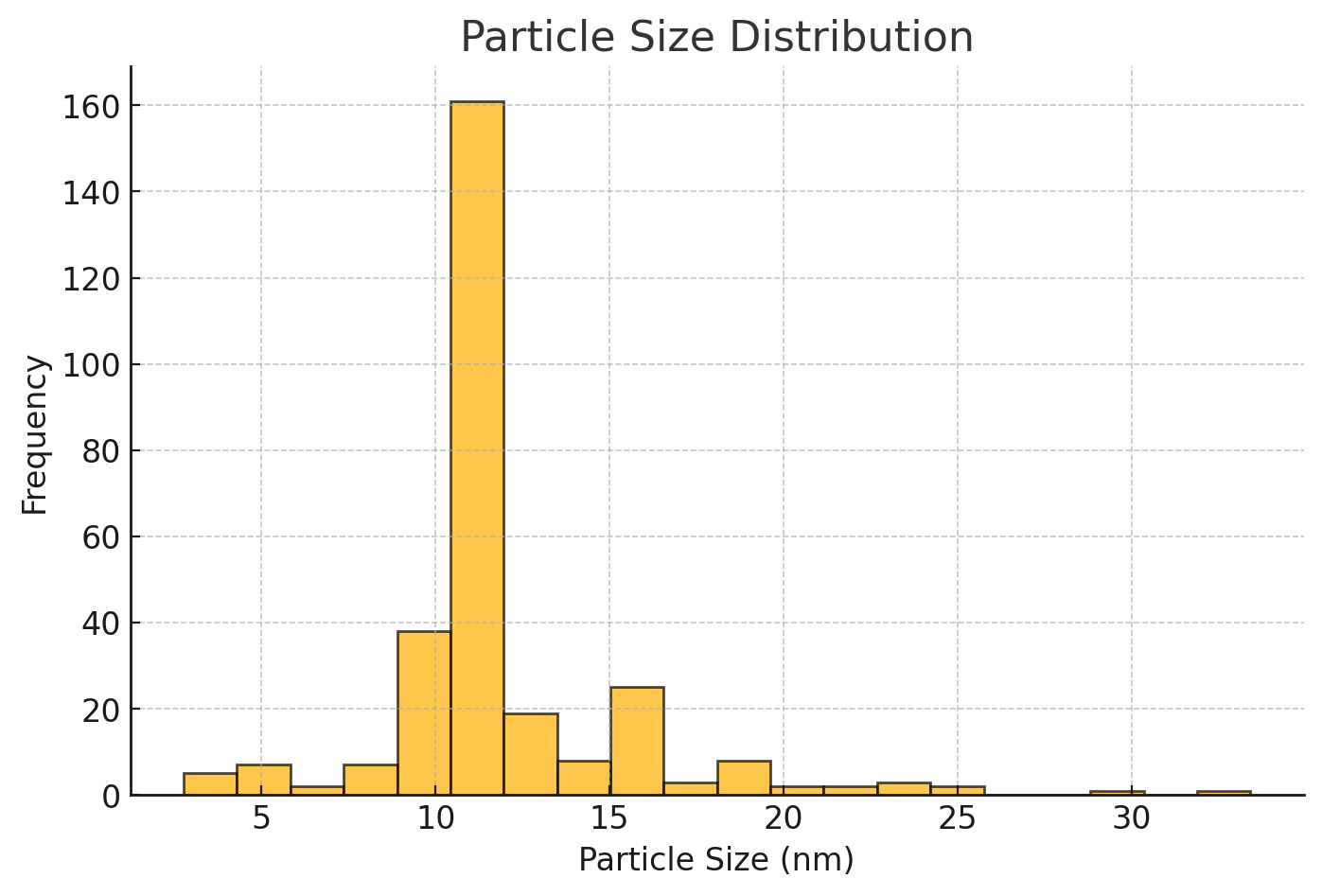


**Figure S2**. Particle size distribution histogram of synthesized AuNPs analyzed using the imageJ software. Particle diameters were extracted (12.0 ± 0.2 nm) using Howard’s method after image enhancement (contrast adjustment) and automatic thresholding Otsus method from TEM images (**Fig. 1c**).


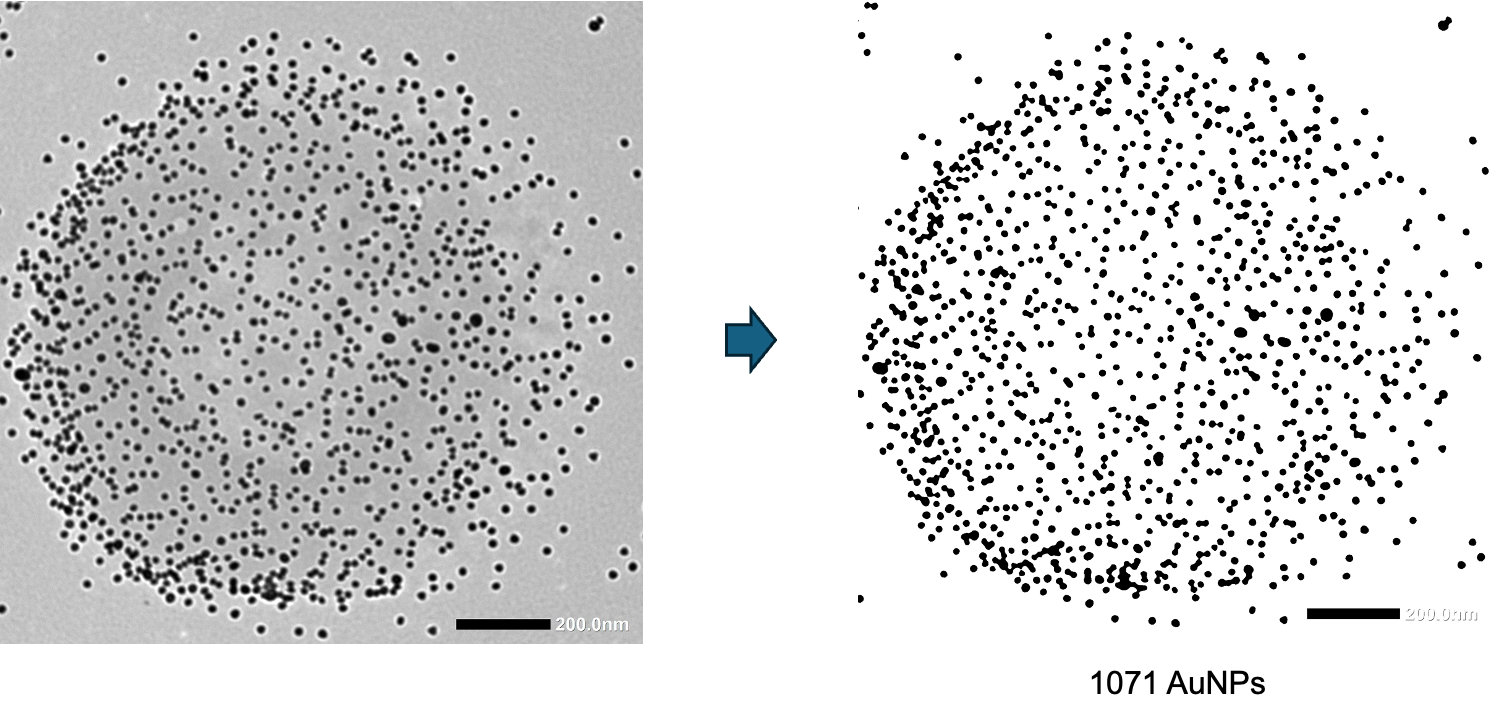


**Figure S3**. TEM image illustrating AuNP localization and counting within a single hybrid microgel (**Fig. 2d**). AuNP counting was performed using ImageJ software with Howard’s method after contrast enhancement and automatic thresholding (Otsu method).


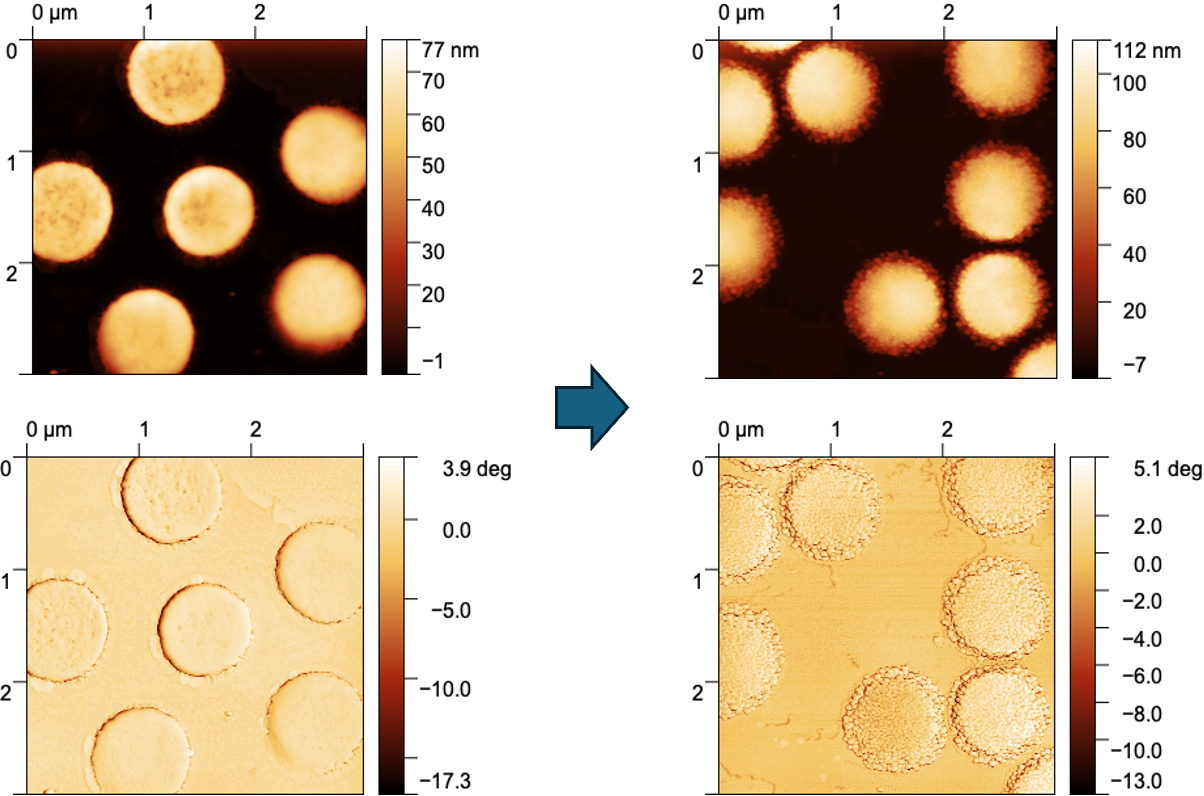


**Figure S4**. AFM height profiles (top row) and phase contrast images (bottom row) of microgels modified with 12.5 mol% SH groups (left column), and HMGs after incorporation of AuNPs (right column). Images demonstrate morphological changes and nanoparticle distribution after AuNP integration.


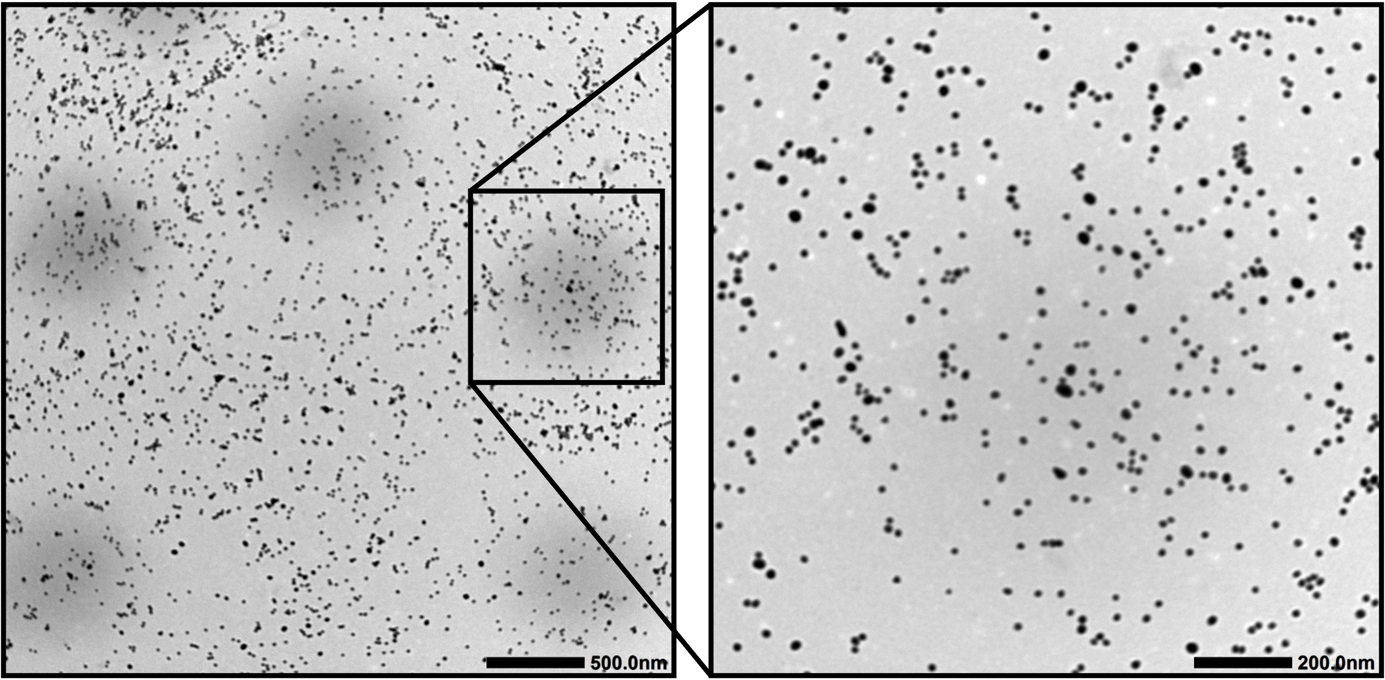


**Figure S5**. TEM image of HMGs without cysteamine modification. The inset shows a magnified view of a single microgel containing AuNPs.


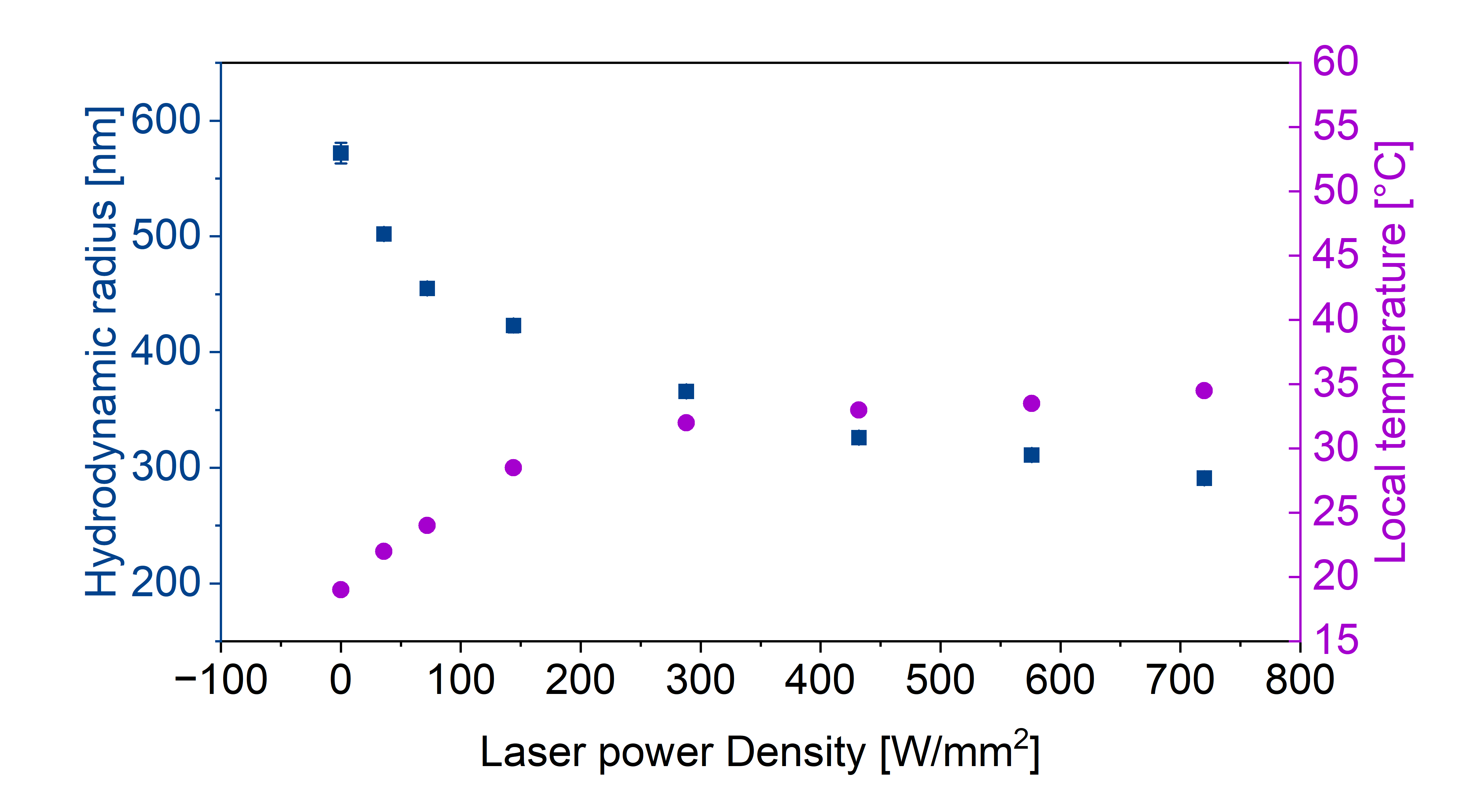


**Figure S6**. Estimation of R_h_ and local temperature for HMGs obtained from dual-laser dynamic light scattering (DLS), under the assumption that the ambient temperature and thus viscosity surrounding each HMG changes in direct accordance with the HMG’s internal temperature.


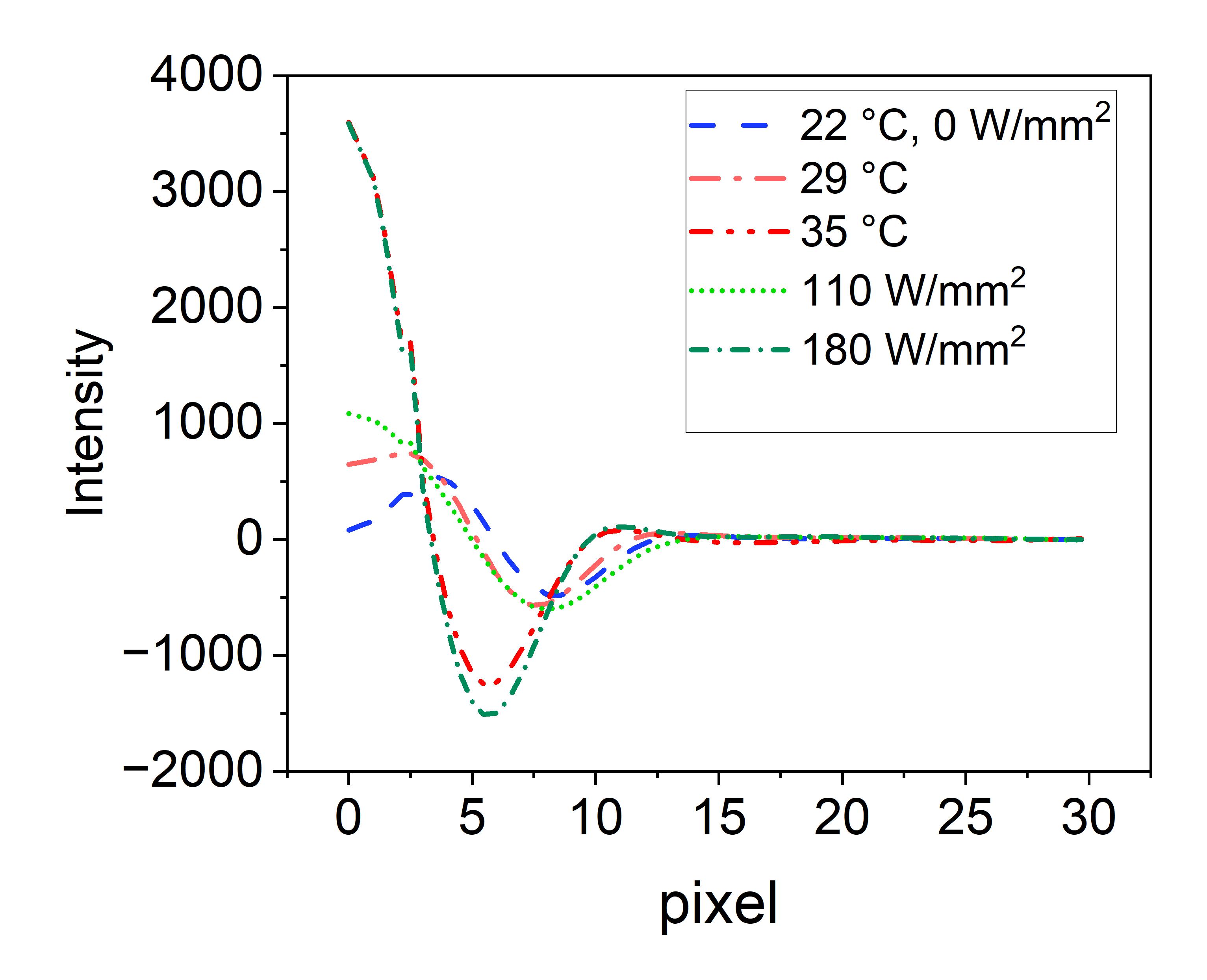


**Figure S7**. Radial intensity profiles obtained via image analysis of HMGs. Representative examples are shown at different temperatures (calibrated via T-dependent batch measurements) and varying green laser powers, illustrating how the intensity gradients evolve under these experimental conditions.


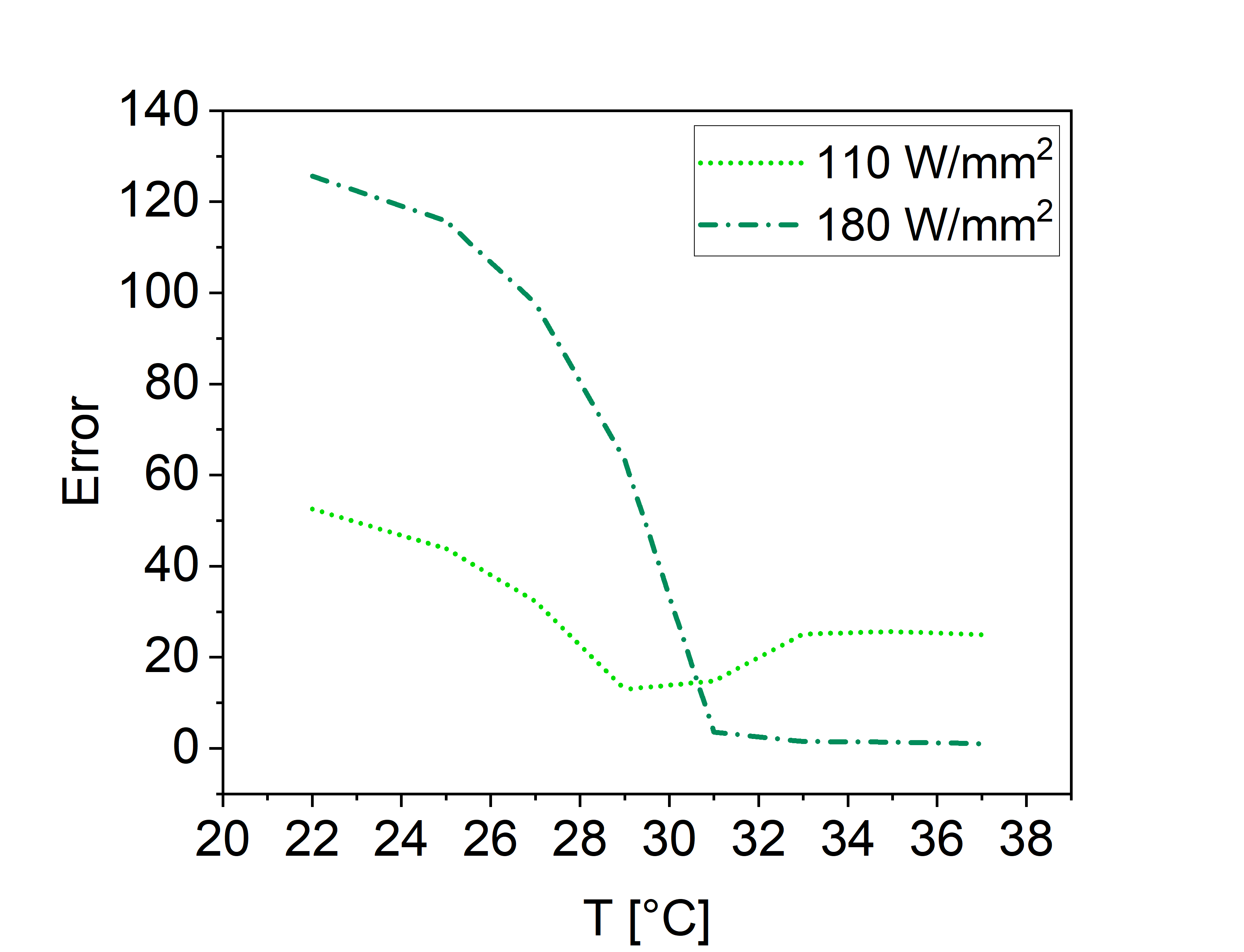


**Figure S8**. Calibration-based estimation of local temperature under plasmonic heating. Reference images of HMGs were first acquired at different known temperatures to construct a calibration dataset. Subsequently, experimental images were recorded under two different 532 nm laser illumination intensities (110 and 180 W/mm^2^). For each condition, the experimental image was compared to the set of calibration images, and the error was computed as a function of temperature. The estimated temperature corresponds to the calibration temperature that minimizes this error. The plot shows the error versus temperature for both laser intensities. For 110 W/mm^2^, a clear minimum is observed near T= 29 °C, indicating a well-defined temperature estimate. In contrast, the 180 W/mm^2^ condition shows relatively flat error values above the VPTT, with no distinct minimum, reflecting the fact that images above the VPTT become visually indistinguishable and thus difficult to differentiate via this image-based method.


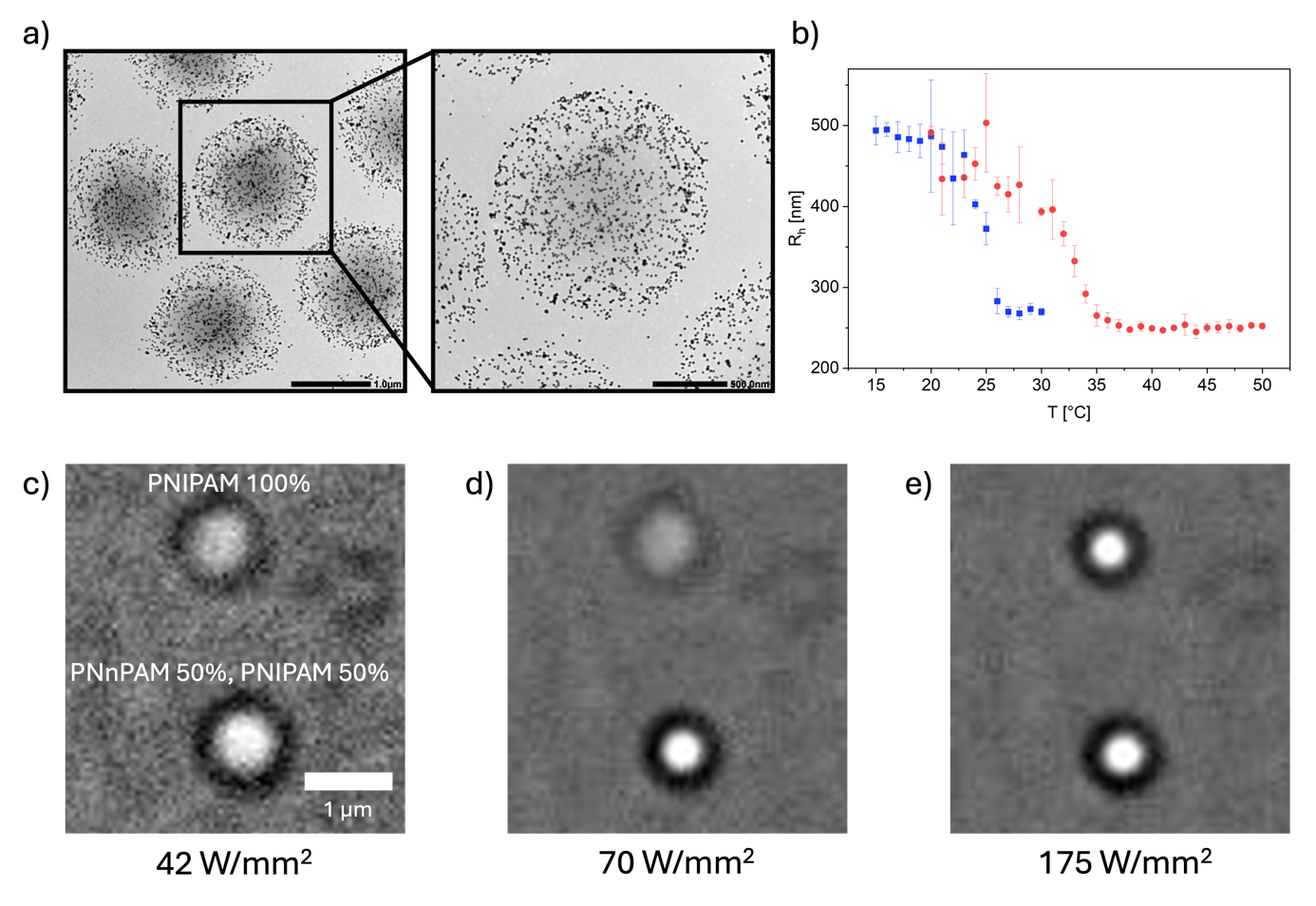


**Figure S9.** (a) TEM images of HMGs composed of 50 mol% PNnPAM and 50 mol% PNIPAM. (b) DLS measurements of both HMG types at different temperatures. Blue square symbols represent HMGs containing 50 mol% PNnPAM and 50 mol% PNIPAM, while red circle symbols correspond to PNIPAM-only HMGs (100 mol% PNIPAM). (c–e) Comparison of the two HMG formulations: PNIPAM 100 mol% and PNnPAM 50 mol% / PNIPAM 50 mol%, under different irradiation conditions. The ambient temperature during optical tweezer measurements was 20 °C. All DLS and optical tweezer measurements were performed at pH 4 buffer (ionic strength 10 mM).

(1) Turkevich, J.; Stevenson, P. C.; Hillier, J. A study of the nucleation and growth processes in the synthesis of colloidal gold. *Discuss. Faraday Soc.* **1951**, *11* (0), 55-75. DOI: 10.1039/DF9511100055.

(2) Liu, J.; Lu, Y. Preparation of aptamer-linked gold nanoparticle purple aggregates for colorimetric sensing of analytes. *Nat. Protoc.* **2006**, *1* (1), 246-252. DOI: 10.1038/nprot.2006.38.

(3) Perrin, D. D. Buffers of Low Ionic Strength for Spectrophotometric pk Determinations. *Aust. J. Chem.* **1963**, *16*, 572-578, DOI: 10.1072/CH9630572.
